# Environmentally friendly Pd(II) recovery from spent automotive catalysts using resins impregnated with a pincer-type extractant

**DOI:** 10.1038/s41598-020-79614-2

**Published:** 2021-01-11

**Authors:** Manabu Yamada, Shun Kimura, Muniyappan Rajiv Gandhi, Atsushi Shibayama

**Affiliations:** 1 Research Center of Advanced Materials for Breakthrough Technology, Graduate School of Engineering Science, Akita University, 1-1 Tegatagakuen-machi, Akita, 010-8502 Japan; 2 Applied Chemistry Course, Department of Materials Science, Graduate School of Engineering Science, Akita University, 1-1 Tegatagakuen-machi, Akita, 010-8502 Japan; 3 Quality Control Department, Panipat Refinery and Petrochemical Complex, Indian Oil Corporation Limited, Haryana, 132140 India; 4 Graduate School of International Resources Science, Akita University, 1-1 Tegatagakuen-machi, Akita, 010-8502 Japan

**Keywords:** Chemistry, Environmental chemistry

## Abstract

Extractant-impregnated resins have potential for recovering platinum group metals selectively and efficiently. Herein, 1,3-bis(2-(octylthio)propan-2-yl)benzene (**1**), a pincer-type extractant, was impregnated in Amberlite XAD-7 resin (**1**-EIR), and the batch Pd(II) sorption conditions, including impregnated amount, shaking time, Pd(II) concentration, HCl concentration, and Pd(II) desorption reagents, were optimized. The maximum Pd(II) sorption capacity of **1**-EIR was 49 mg g^−1^ after 24 h in a 700 ppm Pd(II) solution. Over 20 adsorption–desorption cycles, **1**-EIR showed good reusability, with a sorption percentage (*S*%) of > 92%. However, not all Pd(II) was desorbed from **1**-EIR. Complete Pd(II) collection was achieved by combining desorption with flaking of the Pd–extractant complex from Pd(II)-loaded **1**-EIR by Soxhlet extraction, as ~ 13 mg g^−1^ remained after the 20th adsorption–desorption cycle by absorptiometric method. The sorption mechanism was elucidated based on the Langmuir isotherm model, thermodynamic parameters, and sorption kinetics. Pd(II) sorption by **1**-EIR was spontaneous and endothermic, and the sorption kinetics followed a pseudo-second-order model. Notably, **1**-EIR also exhibited high selectivity for Pd(II) from a simulated mixed metal solution and a spent automotive catalyst leachate (*S*% = 98% and > 99%, respectively). Thus, this extractant-impregnated system is promising for selective Pd(II) recovery from spent automotive catalysts and other secondary resources.

## Introduction

The demand for Pd, a platinum group metal (PGM), is high because PGMs are essential for improving the performance of automotive catalysts to meet exhaust gas regulations^1^. As the use of exhaust catalysts for motorcycles has recently been implemented in India, increased Pd(II) usage is inevitable^2^. Secondary resources, including spent automotive catalysts, industrial catalysts, and electronic scraps, are a main source of Pd^3,4^. Approximately 60% of Pd produced globally is used in automotive catalytic converters^1^, and generally, the PGM concentrations in automotive catalysts are higher than those in PGM ores. Therefore, the development of effective methods for separating Pd from spent products is key in terms of resource recycling and sustainability.

Generally, in hydrometallurgy, liquid–liquid extraction is used to isolate and purify PGMs from primary and secondary resources because large amounts of metals can be separated^5–7^. For Pd(II), di-*n*-alkyl sulfides (DAS) such as di-*n*-hexyl sulfide and di-*n*-octyl sulfide are well known as good extractants^5,8^. Although DAS can selectively separate Pd(II) from Pd(II)/Pt(IV) mixtures, the Pd(II) extraction kinetics are slow and the sulfide group can be oxidized by oxidants in the acidic aqueous phase^9,10^.

To overcome the disadvantages of DAS, we designed 1,3-bis(2-(octylthio)propan-2-yl)benzene (**1**), which has thioether moieties at the 1- and 3-positions of benzene, as a “pincer-type extractant”^11^. Extractant **1** exhibits high stability in strong acids such as 12 M (M = mol dm^-3^) HCl, 7 M HNO_3_, and HCl/HNO_3_; therefore, **1** is not decomposed or oxidized when exposed to such acids for 7 days. In addition, highly selective and effective Pd(II) recovery from single Pd(II) solutions in acidic media (HCl, HNO_3_, or HCl/HNO_3_), solutions containing multiple metals, and leachates of automotive catalysts has been achieved using pincer-type extractant **1**. Interestingly, the pincer-type extractant bonds specifically to Pd(II) via sulfur–carbon–sulfur (SCS) coordination by releasing a proton from the aromatic moiety of the ligand, which becomes a mono-anion species. This SCS coordination to the metal ion results in high selectivity for Pd(II) from Pd(II)-containing solutions through liquid–liquid extraction. Furthermore, the reusability of pincer-type extractant **1** in organic phases has been confirmed.

Although liquid–liquid extraction is an effective method in hydrometallurgy for separating large amounts of metals, large volumes of organic solvent are also required for extractant dilution, which poses a risk to humans and the environment in terms of toxicity and explosiveness. In industrial processes, explosion-proof measures must be adopted to ensure safety. Thus, it is costly to implement new solvent extraction processes in industrial plants.

Adsorption by ionic exchange resins is another separation technique that can be used for PGM recovery^12–15^. The use of adsorbents for metal recovery has several merits: (1) adsorbents are easy-to-handle and easy-to-treat before and after adsorption, (2) adsorption processes can easily be introduced into industrial plants, and (3) diluents such as harmful organic solvents are not required. Thus, the development of new adsorbents for metal separation is ongoing^15–17^. Extractant-impregnated resins (EIRs) are of particular interest because they allow the properties of extractants to be incorporated into an adsorbent. Recently, to reduce organic solvent use, we have focused on preparing EIRs as adsorbents using a thiacarbamoyl- or diethylphosphate-modified thiacalixarene as an extractant with Amberlite XAD-7 as a base resin^18,19^. The two EIRs show high selectivity for Pd(II) from a solution containing 11 metals or an automotive catalyst leachate^18,19^. Among resins, the nonionic polymer Amberlite XAD-7 has good qualities for metal adsorption owing to its macroreticular structure, high porosity, high surface area, and excellent chemical stability (Fig. S1 and Table S1).

Herein, we developed an EIR based on XAD-7 impregnated with pincer-type extractant **1** (**1**-EIR). We investigated the Pd(II) adsorption properties of **1**-EIR using surface analysis, sorption isotherms, and sorption kinetics. Further, the effectiveness of various processes for stripping Pd(II) from Pd(II)-loaded **1**-EIR was evaluated. In addition, as a practical example, **1**-EIR was applied for Pd(II) recovery from a spent automotive catalyst leachate containing 10 different metals, including PGMs, base and minor metals, and rare earth metals.

## Results and discussion

### Preparation of 1-EIR

**Figure 1:**
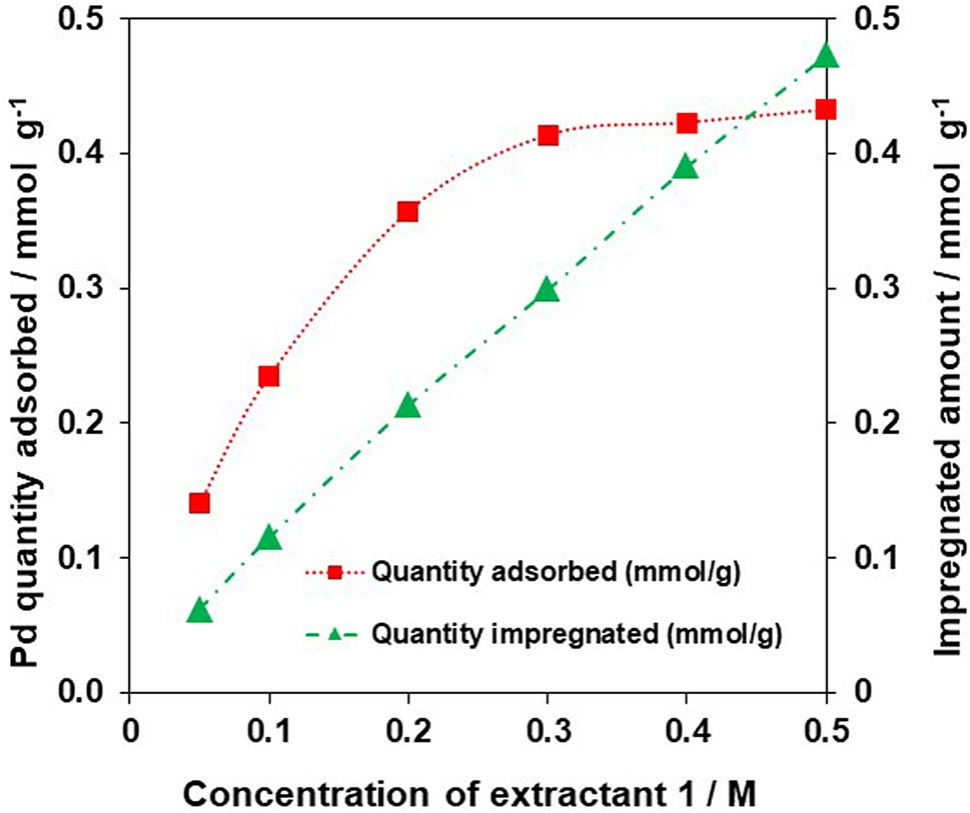
Relationship between amount of **1** impregnated in XAD-7 and the Pd(II) quantity adsorbed by **1**-EIR. Sorption conditions: **1**-EIR = 0.1 g; [Pd(II)] = 500 ppm (= mg L^−1^); [HCl] = 0.1 M; shaking speed = 280 rpm; shaking time = 24 h.

Figure 1 shows the relationship between the amount of **1** impregnated in XAD-7 and the amount of Pd adsorbed by **1**-EIR. As the concentration of **1** in acetone increased, the impregnated amount increased linearly. In contrast, the quantity of adsorbed Pd(II) reached saturation when the concentration of **1** in acetone was ~ 0.3 M. This behavior suggested that **1** formed a monolayer on the surface of XAD-7 during impregnation at concentrations up to 0.3 M, but a multilayer was formed at higher concentrations. Under the optimized impregnation conditions (0.3 M extractant **1**, immersion time of 24 h), the impregnated amount was 130 mg g^−1^ (0.288 mmol g^−1^).

### Effect of HCl concentration on Pd(II) adsorption

We investigated the effect of various HCl concentrations (0.1–8.0 M) on Pd(II) adsorption by **1**-EIR (Fig. S2). No significant change in the Pd(II) adsorption efficiency was observed in the investigated HCl concentration range. This result is consistent with our previous findings for liquid–liquid extraction using **1** at various HCl concentrations^11^. Thus, the effectiveness of **1**-EIR for Pd(II) adsorption is likely due to the Pd(II) extraction ability of **1**.

### Effect of shaking time on Pd(II) adsorption

The effect of the shaking time (0–240 min) on Pd(II) adsorption by **1**-EIR was also investigated (Fig. S3). At shaking times of 50 and 75 min, the Pd(II) sorption percentages (*S*%) were 81.5% and 90.0%, respectively. The adsorbed quantity of Pd(II) reached saturation (*S*% = 99%) at shaking times longer than 150 min.

### Pd(II) desorption from Pd(II)-Loaded 1-EIR

The desorption of Pd(II) from Pd(II)-loaded **1**-EIR was investigated using various stripping reagents (Table 1). The most effective combination of stripping reagent with acids or bases was the CH_4_N_2_S/HCl system, which exhibited Pd(II) desorption percentages (*D*%) values of 56.6% and 65.6% with 0.1 and 1.0 M CH_4_N_2_S, respectively. In contrast, ammonia-containing systems (CH_4_N_2_S/NH_4_OH and NH_4_Cl/HCl) showed ineffective desorption of Pd(II) (*D*% = 0.6% and 0.2%, respectively). Similarly, Na_2_SO_3_/H_2_O and Na_2_S_2_O_3_/H_2_O systems gave low *D*% values of 2.7% and 23.1%, respectively. Hence, as the optimal Pd(II) stripping reagent, 1.0 M CH_4_N_2_S/1.0 M HCl system was utilized in any subsequent desorption processes.

**Table 1:** Pd(II) desorption percentage (*D*%) using stripping reagents combined with acids or bases.

Stripping reagents	*D*%
0.1 M CH_4_N_2_S/1.0 M HCl	56.6
1.0 M CH_4_N_2_S/1.0 M HCl	65.6
1.0 M CH_4_N_2_S/2.8 M NH_4_OH	0.6
0.1 M NH_4_Cl/1.0 M HCl	0.2
1.0 M Na_2_SO_3_/H_2_O	2.7
1.0 M Na_2_S_2_O_3_/H_2_O	23.1

### Reusability of 1-EIR

**Figure 2:**
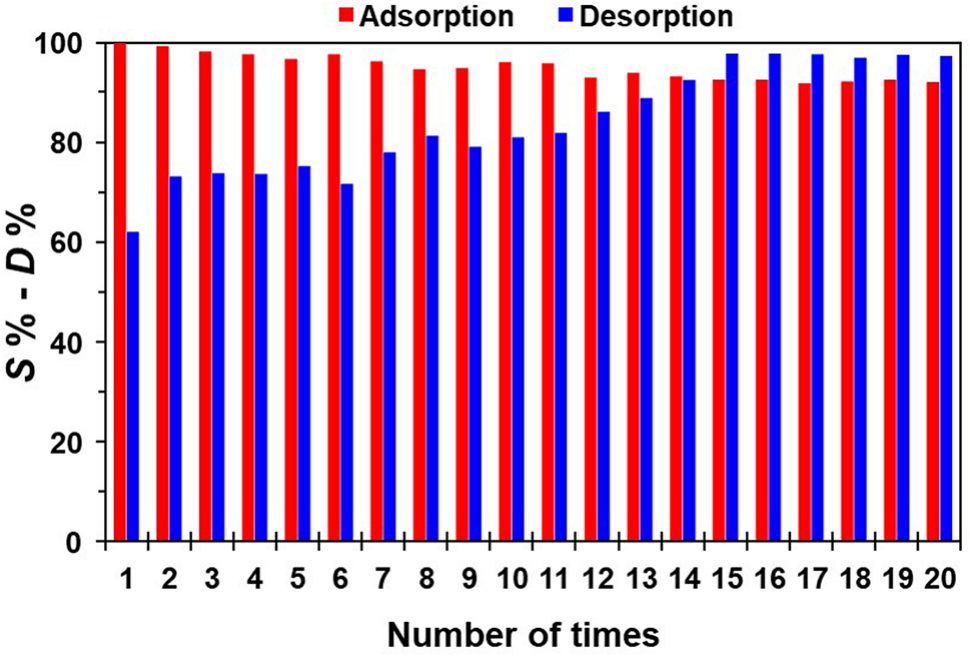
Pd(II) adsorption and desorption cycles using **1**-EIR. Adsorption conditions: [**1**-EIR] = 0.1 g; [Pd(II)] = 100 ppm (= mg L^−1^); [HCl] = 0.1 M; shaking speed = 280 rpm; shaking time = 3 h. Desorption conditions: **1**-EIR = 0.1 g; [CH_4_N_2_S] = 1.0 M; [HCl] = 1.0 M; shaking speed = 280 rpm; shaking time = 1 h.

The reusability of **1**-EIR was evaluated over repeated Pd(II) adsorption and desorption cycles (Fig. 2). The adsorption of Pd(II) by **1**-EIR was effective, with an *S*% value of ~ 99% for each cycle. In contrast, the desorption of Pd(II) from Pd(II)-loaded **1**-EIR exhibited slightly different behavior, as the initial *D*% value of 65.6% gradually increased, reaching a constant value of > 97% after 15 cycles. Although **1**-EIR adsorbed Pd(II) effectively and completely at various HCl concentrations, the complete desorption of Pd(II) from Pd(II)-loaded **1**-EIR was not achieved using the surveyed stripping reagents. This result suggests that undesorbed Pd(II) is located deep within **1**-EIR, where access by CH_4_N_2_S molecules is inhibited by insufficient space between Pd(II)-sorbed sites in the pores. The subsequent improvement in Pd(II) desorption efficiency may be due to CH_4_N_2_S molecules reaching Pd(II) adsorbed in wider pores of **1**-EIR because the narrower pores were increasingly stacked with undesorbed Pd(II) from previous cycles. After 20 adsorption–desorption cycles, the calculated amount of Pd(II) remaining in **1**-EIR was ~ 31 mg g^−1^. As discussed below, the reason that some Pd(II) remains in **1**-EIR after the desorption process can be explained by the specific area and pore size distribution. In addition, the hypothesis that Pd(II) sorption occurs on the surface and inside the pores of **1**-EIR can be confirmed theoretically by the sorption dynamics.

### Characterization of Pd(II)-loaded 1-EIR

#### Digital images and SEM–EDX studies

To indirectly and directly elucidate whether Pd(II) was adsorbed by **1**-EIR, unloaded **1**-EIR and Pd(II)-loaded **1**-EIR were evaluated by digital photography and SEM–EDX. As shown in Fig. S4, the surface of **1**-EIR was colorless before Pd(II) adsorption but light brown after Pd(II) sorption, indicating that Pd coated the surface of **1**-EIR. Similarly, the color of the Pd(II) solution changed from brown to pale brown after Pd(II) adsorption by **1**-EIR. From the observed color changes, it is suggested that **1**-EIR adsorbed Pd(II) from the Pd(II) solution.

**Figure 3:**
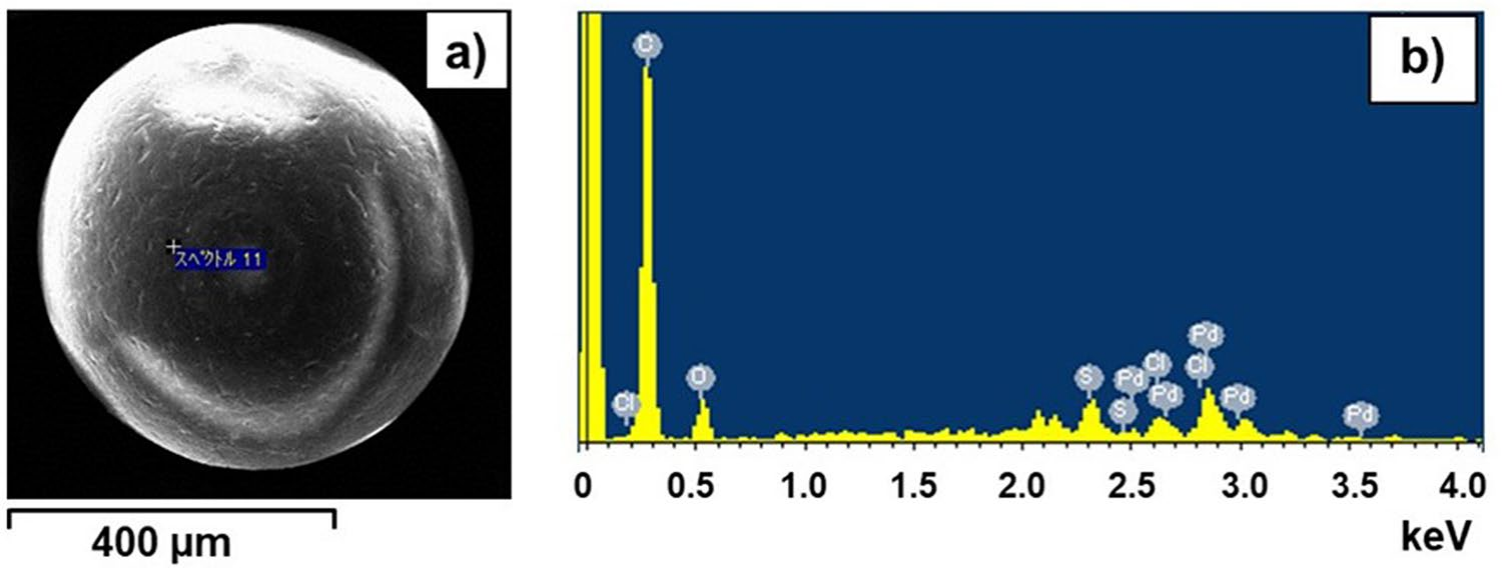
(**a**) SEM image and (**b**) EDX spectrum after Pd(II) sorption by **1**-EIR.

As direct evidence for Pd(II) sorption on **1**-EIR, Figs. 3a, b show an SEM image and an EDX spectrum after Pd(II) sorption by **1**-EIR. After Pd(II) adsorption, peaks corresponding to C Kα (0.22 keV), O Kα (0.52 keV), S Kα (2.3 keV), Cl Kα (2.6 keV), and Pd Kα (2.8 keV) were observed in the EDX spectrum, clearly showing the presence of Pd(II) in the **1**-EIR matrix.

### XPS studies

**Figure 4:**
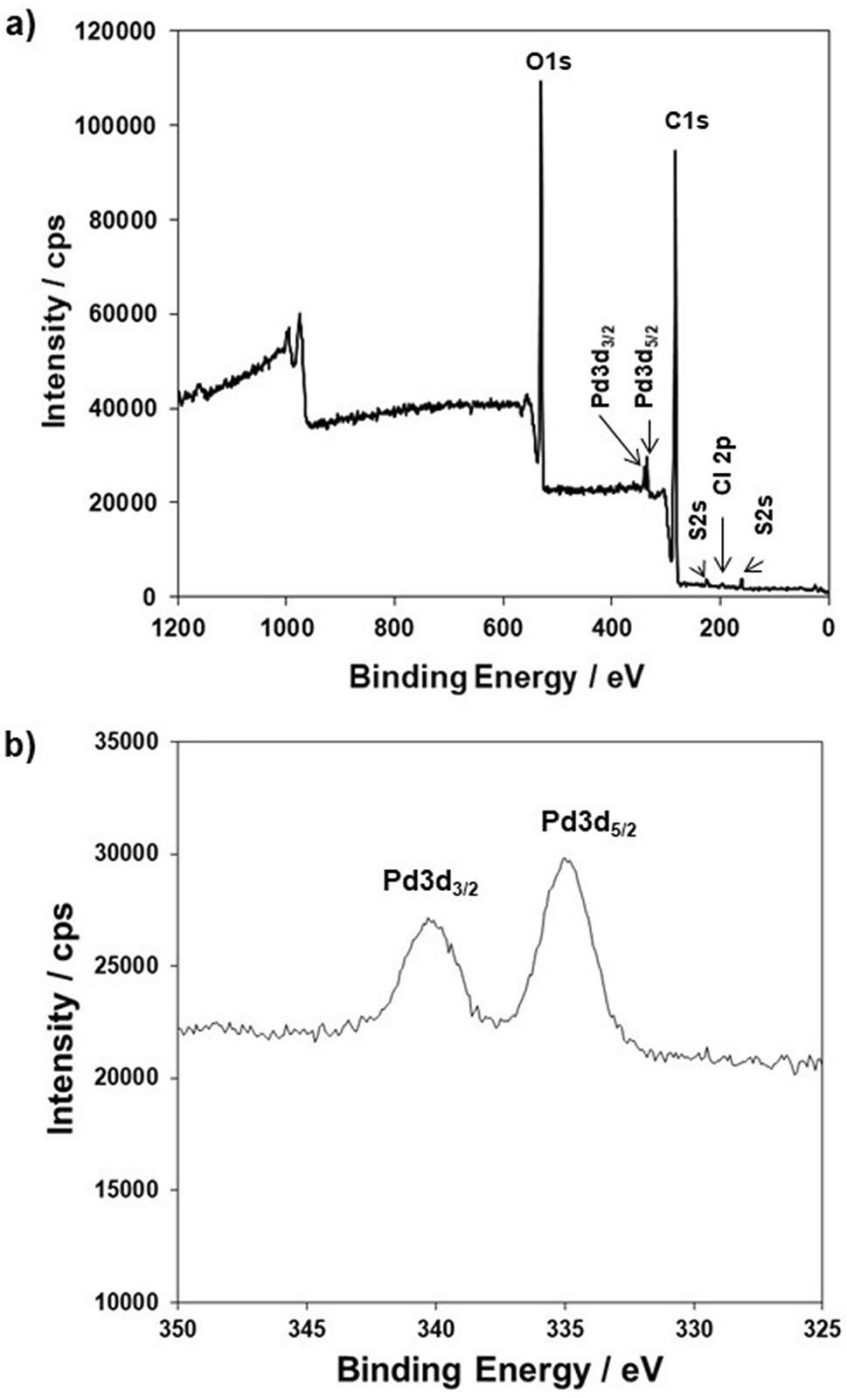
(**a**) Full-scan and (**b**) Pd 3d XPS spectra of Pd(II)-loaded **1**-EIR after Pd(II) adsorption.

XPS was performed to elucidate the chemical or electronic state of each element on the surface of Pd(II)-loaded **1**-EIR. The full-scan XPS spectrum for Pd(II)-loaded **1**-EIR (Fig. 4a) confirmed the presence of C, S, O, Cl, and Pd. Large C 1*s* and O 1*s* peaks derived from methyl (CH_3_–), methylene (–CH_2_–), aromatic (C=C and C–C), quaternary carbon (=C=), and ester (–C(=O)–O–) moieties were observed at 285 and 531 eV, respectively. Small S 2*p* and S 2*s* peaks arising from sulfide (–S–) groups appeared at 164 and 228 eV, respectively. The Cl 2*p* peak at 200 eV indicated the Cl was coordinated to adsorbed Pd(II) during sorption. In the high-resolution Pd 3*d* XPS spectrum (Fig. 4b), Pd 3*d*_3/2_ and Pd 3*d*_5/2_ peaks derived from Pd(II) species adsorbed on **1**-EIR were observed at 340 and 335 eV, respectively. These observations are consistent with the EDX results and confirm that the presence Pd(II) in the **1**-EIR matrix following sorption.

### Specific surface area and pore size distribution

To clarify why Pd(II) remained in **1**-EIR after Pd(II) stripping, the specific surface areas and pore size distributions of XAD-7, **1**-EIR, and Pd(II)-loaded **1**-EIR were determined from the nitrogen adsorption isotherms. All the nitrogen adsorption isotherms (Fig. S5) were type II, indicating that a multilayer adsorption process occurred. However, the quantity of nitrogen adsorbed on XAD-7 was two times higher than that on **1**-EIR, which suggests that the impregnation of **1** had a significant effect on the adsorption properties of XAD-7. Similarly, the BET specific surface areas of XAD-7 and **1**-EIR were 501 and 254 m^2^ g^−1^ (Fig. S6), which can be attributed to the packing of extractant **1** into the pores of XAD-7. In fact, impregnation with the extractant even led to the blockage of specific pores in the resin. This result was supported by the observed pore size distributions (Fig. S7). XAD-7, **1**-EIR, and Pd(II)-loaded **1**-EIR all exhibited pore sizes in the range of 1.2–19.2 nm, with the most common pore size being 1.2 nm and the proportion gradually decreasing with increasing pore size. However, the pore size distributions before and after impregnation of **1** in XAD-7 were clearly different. In particular, the d*V*_p_/d*r*_p_ value for pore sizes of 1.2–3.5 nm decreased (~ 26%) after impregnation, i.e., the above pore size region was blocked and/or reduced by impregnating extractant **1** on XAD-7. As displayed in Fig. S8, PdCl_4_^2−^ (size: ~ 4.4 Å) can approach extractant **1** in pores of ~ 1.2 nm, and a CH_4_N_2_S molecule (~ 4.0 × 3.6 Å) as a stripping reagent can also reach the Pd(II)–extractant **1** complex in such pores. However, the stripping of Pd(II) from the Pd(II)–extractant **1** complex requires two CH_4_N_2_S molecules and one Cl^−^ ion. Therefore, this process is very difficult in small pores because there is not sufficient space to form the PdCl_2_(CH_4_N_2_S)_2_ species.

### Complete collection of Pd(II) via flaking

To achieve desorption of residual Pd(II) in **1**-EIR, the Pd(II)–extractant **1** complex was extracted using an organic solvent was selected. After Soxhlet extraction for 12 h in methanol, the resin was colorless and solution changed from colorless to pale yellow (Fig. S9). XPS measurements were performed to confirm that Pd(II) was completely removed by this process (Fig. S10). The full-scan XPS exhibited two peaks at 530 and 282 eV corresponding to O 1*s* and C 1*s*, respectively, derived from the structure of XAD-7. As no S, Pd, and Cl peaks were observed, Pd(II) and pincer-type extractant **1** were not detected by XPS, which indicates that the complete collection of Pd(II) was achieved via flaking of the Pd(II)–extractant complex by Soxhlet extraction using methanol. The Pd(II) amount in the extracted Pd(II) solution was ~ 13 mg g^-1^ by measuring absorbance determination in absorption wavelength of Pd-extractant **1** complex at 311 nm. Thus, it is proposed that **1**-EIR is an excellent Pd(II) adsorbent with high reusability and Pd(II) remaining in **1**-EIR can be easily recovered by extraction with methanol.

### Mechanism of Pd(II) sorption by 1-EIR

#### ^1^H NMR study

**Figure 5:**
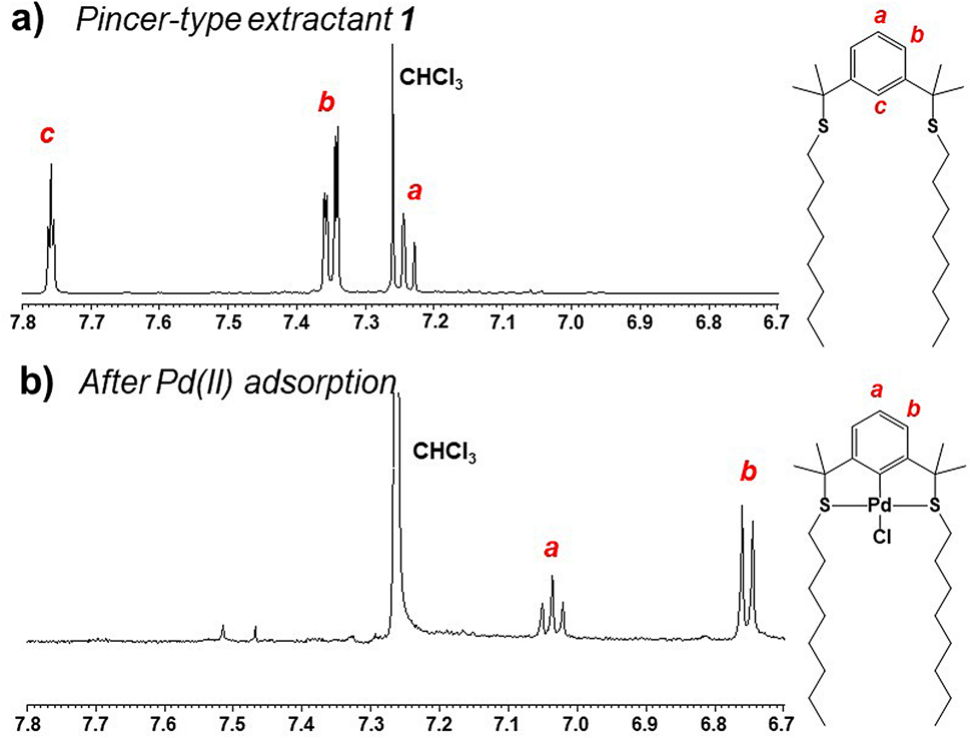
Partial ^1^H NMR spectra of (**a**) pincer-type extractant **1** and (**b**) the Pd(II)–extractant **1** complex flaked from Pd(II)-loaded **1**-EIR (500 MHz, CDCl_3_, δ from TMS).

An understanding of the Pd(II) adsorption mechanism is fundamental for metal separation and industrial development. We used ^1^H NMR to investigate the mechanism of Pd(II) sorption by **1**-EIR (Fig. S11). Figures 5a, b show the partial ^1^H NMR spectra of extractant **1** and the Pd(II)–extractant **1** complex collected by extraction from Pd(II)-loaded **1**-EIR using chloroform. In the ^1^H NMR spectrum of extractant **1**, three chemical shifts were observed in the aromatic region at 7.24 ppm (*a*), 7.35 ppm (*b*), and 7.76 ppm (*c*). In contrast, the ^1^H NMR spectrum of the Pd(II)–extractant **1** complex, the aromatic protons exhibited only two chemical shifts of 7.03 ppm (*a*) and 6.75 ppm (*b*). Thus, after complexation with Pd(II), the peak observed for **1** at 7.76 ppm disappeared, whereas the other two peaks shifted upfield owing to the effect of Pd in the complex. Thus, upon uptake of Pd(II), in addition to SCS coordination, the aromatic proton at *c* in the structure of **1** was released. Thus, it is suggested that Pd(II) was adsorbed via metalation by the SCS elements of extractant **1** in the impregnated resin. This result is similar to the Pd(II) extraction mechanism of **1** during solvent extraction reported by our group^11^.

### Sorption isotherms

#### Loading capacity of Pd(II) on **1**-EIR at various temperatures

**Figure 6:**
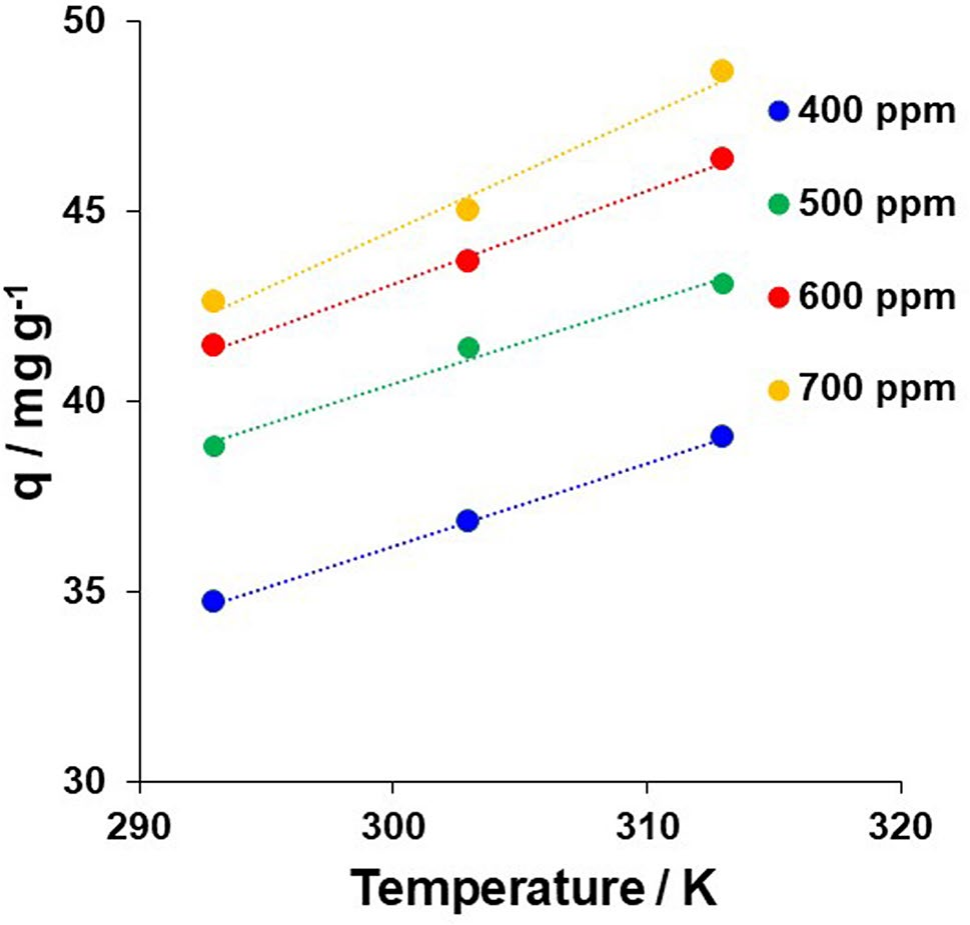
Effect of temperature on Pd(II) sorption by **1**-EIR at various Pd(II) concentrations. Sorption conditions: **1**-EIR = 0.1 g, [HCl] = 0.1 M; [Pd(II)] = 400–700 ppm (= mg L^−1^); temperature = 293–313 K; shaking time = 24 h; shaking speed = 80 rpm.

To investigate the sorption isotherm model, the effect of temperature (293–313 K) on the Pd(II) loading capacity of **1**-EIR was evaluated using 400, 500, 600, and 700 ppm Pd(II) solutions (Fig. 6). The sorption capacity (*q* (mg g^−1^)) of **1**-EIR was proportional to the temperature, with the maximum *q* values observed at 293, 303, and 313 K being 43, 45, and 49 mg g^−1^, respectively.

#### Isotherm models

To quantify the sorption capacity of **1**-EIR, the Pd(II) sorption data were evaluated using the two most common isotherm models, namely, the Freundlich and Langmuir isotherms.

The linear form of the Freundlich isotherm^21^ is represented by Eq. (1):

1 $$ {\text{log}}q_{{e{ }}} = {\text{log}}k_{f} + { }\frac{1}{n}{\text{log}}C_{{e{ }}} $$

where *q*_*e*_ is the amount of Pd(II) adsorbed per unit mass of the resin (mg g^−1^), *C*_*e*_ is the equilibrium concentration of Pd(II) in solution (mg L^−1^), *k*_*f*_ is a measure of the adsorption capacity, and 1/*n* is the adsorption intensity. The Freundlich isotherms for **1**-EIR are presented in Fig. S12a, and the corresponding Freundlich isotherm parameters are listed in Table 2. The log *q*_*e*_ versus log *C*_*e*_ plots were linear, as indicated by the high correlation coefficient (*r*) values, which indicates the applicability of the Freundlich isotherm model. The *n* values were between 1 and 10, showing that Pd(II) adsorption is a favorable process^22^. Further, the *k*_*f*_ values increased with increasing temperature, indicating that Pd(II) sorption by **1**-EIR is an endothermic process.

**Table 2:** Freundlich and Langmuir isotherm parameters for Pd(II) adsorption by **1**-EIR.

Temp. (K)	Freundlich			Langmuir		
	*n*	*k*_*f*_ (mg g^−1^)(L mg^−1^)^1^^/n^	*r*	*Q*º (mg g^−1^)	*b* (L g^−1^)	*r*
293	8.9286	24.24	0.961	43.860	0.0919	0.999
303	9.1408	25.34	0.990	47.847	0.2330	0.999
313	9.8232	29.43	0.971	51.282	− 0.0301	0.998

The Langmuir isotherm model^23^ is represented by Eq. (2):

2 $$ \frac{{C_{e} }}{{q_{{e{ }}} }} = \frac{1}{{Q^{0} b}} + { }\frac{{C_{e} }}{{Q^{0} }} $$

where *Q*º is the amount of adsorbate at complete monolayer coverage (mg g^−1^), from which the maximum sorption capacity of the sorbent can be derived, and *b* (L mg^−1^) is the Langmuir isotherm constant, which is related to the energy of adsorption^24^. The Langmuir isotherms for **1**-EIR are shown in Fig. S12b, and the corresponding Langmuir isotherm parameters are listed in Table 2. The *r* values of the linear *C*_*e*_/*q*_*e*_ versus *C*_*e*_ plots were high, which indicates the applicability of the Langmuir isotherm model. Further, the values of *Q*º increased with increasing temperature, suggesting that Pd(II) sorption by **1**-EIR was endothermic.

Based on these evaluations of the Freundlich and Langmuir isotherms, it can be suggested that Pd(II) adsorption by **1**-EIR followed the Langmuir mechanism because the *r* values are slightly higher than that of Freundlich isotherm and that Pd(II) was adsorbed by a single-layer adsorption process.

### Thermodynamics of the sorption process

The thermodynamic parameters associated with adsorption (i.e., the standard free energy change (Δ*G*º), standard enthalpy change (Δ*H*º), and standard entropy change (Δ*S*º)) were calculated as follows. The free energy of the sorption process is given by Eq. (3):

3 $$ \Delta G^{0} = { } - {\text{R}}T{\text{ln}}K_{{0{ }}} $$

where Δ*G*º is the standard free energy of sorption (kJ mol^−1^), *T* is the temperature (K), R is the universal gas constant (8.314 J mol^−1^ K^−1^), and *K*_0_ is sorption equilibrium coefficient. The *K*_0_ values at different temperatures were determined by extrapolating the slope of the plot of ln(*q*_*e*_/*C*_*e*_) versus *C*_*e*_ to zero *C*_*e*_, according to the method suggested by Khan and Singh^23^. *K*_0_ can be expressed in terms of Δ*H*º and Δ*S*º as a function of temperature:

4 $$ {\text{ln}}K_{0} = { }\frac{{\Delta S^{0} }}{{\text{R}}} - \frac{{\Delta H^{0} }}{{{\text{R}}T}} $$

where Δ*H*º is the standard enthalpy change (kJ mol^−1^) and Δ*S*º is the standard entropy change (kJ mol^−1^ K^−1^). The Δ*H*º and Δ*S*º values can be obtained from the slope and intercept of the plot of ln *K*_o_ versus 1/T (Fig. S13). The negative values of Δ*G*º at all the investigated temperatures (− 11.837, − 12.017, and − 9.4453 kJ mol^−1^ at 293, 303, and 313 K, respectively) confirm the spontaneous nature of Pd(II) sorption by **1**-EIR. The positive Δ*S*º value (46.414 kJ mol^−1^) indicates that the freedom of the Pd(II) ions is not very restricted and that there is an increase in randomness at the solid/solution interface during Pd(II) sorption. The positive Δ*H*º value (0.1166 kJ mol^−1^ K^−1^) confirms that Pd(II) sorption by **1**-EIR is an endothermic process.

### Sorption dynamics

The two main types of sorption kinetic models (i.e., reaction-based and diffusion-based models) were used to fit the experimental data. Knowledge of the batch sorption kinetics is necessary for the design of an industrial adsorption column.

### Reaction-based models

Pseudo-first-order and pseudo-second-order kinetic models were used to investigate the Pd(II) sorption mechanism of **1**-EIR. The simple pseudo-first-order kinetic model^24^ can be represented by the Lagergren equation, as follows:

5 $$ {\text{log}}(q_{e} - q_{t} ) = { }\frac{{k_{ad} }}{2.303}t{ } $$

where *q*_*t*_ is the amount of Pd(II) on the surface of the resin at time *t* (mg g^−1^) and *k*_*ad*_ is the equilibrium rate constant of pseudo-first-order sorption (min^−1^). The *k*_*ad*_ values under different experimental conditions were determined from the linear log(*q*_*e*_ − *q*_*t*_) versus *t* plots (Fig. S14 and Table S2). The linearity of these plots, as indicate by the high *r* values, indicated the applicability of the Lagergren equation.

The pseudo-second-order model is also widely used. Although there are various linear pseudo-second-order kinetic models^25^, the most popular form is represented by Eq. (6):

6 $$ \frac{t}{{q_{t} }} = { }\frac{1}{h} + \frac{t}{{q_{e} }} $$

where *q*_*t*_ = (*q*_*e*_^2^*kt*)/1 + *q*_*e*_*kt* indicates the amount of Pd on the surface of the resin at a given time, *t* (mg g^−1^), *k* is the pseudo-second-order rate constant (g mg^−1^ min^−1^), *q*_*e*_ is the amount of Pd(II) sorbed at equilibrium (mg g^−1^), and *h* is the initial sorption rate (mg g^−1^ min^−1^), where *h* = *kq*_*e*_^2^. The values of *q*_*e*_ (1/slope), *k* (slope^2^/intercept), and *h* (1/intercept) (Table S2) were determined from the linear *t*/*q*_*t*_ versus *t* plots (Fig. S15). The high *r* values of these plots indicated the applicability of the pseudo-second-order model. The *r* values of the pseudo-second-order model were higher than those of the pseudo-first-order model, indicating the greater applicability of the pseudo-second-order model. From the result of reaction-based models, it is possible to evaluate whether Pd(II) sorption is occurring either on the surface or inside the pores of **1**-EIR. In this case, it might be estimated that the Pd(II) sorption by the diffusion process predominantly occurs in the pores of **1**-EIR.

### Diffusion-based models

For a solid–liquid sorption process, solute transfer is usually characterized by either particle diffusion or pore diffusion control. A simple equation for the particle-diffusion-controlled sorption process^26^ is as follows:

7 $$ {\text{ln}}\left( {1 - \frac{{C_{t} }}{{C_{e} }}} \right) = { } - k_{p} t{ } $$

where *k*_*p*_ is the particle diffusion coefficient (mg g^−1^ min^−1^). The value of the particle diffusion coefficient can be obtained from the slope of the ln(1 − *C*_*t*_/*C*_*e*_) versus *t* plot (Fig. S16). The pore (intraparticle) diffusion model used here relies on the theory proposed by Weber and Morris^27^. The intraparticle diffusion equation is given as follows:

8 $$ q_{t} = { }k_{i} t^{\frac{1}{2}} $$

where *k*_*i*_ is the intraparticle diffusion coefficient (mg g^−1^ min^−0.5^). The value of the intraparticle diffusion coefficient^28^ can be obtained from the slope of the *q*_*t*_ versus *t*½ plot (Fig. S17). The calculated parameters for the particle diffusion and pore diffusion models are listed in Table S2. The observed high *r* values suggest that Pd(II) sorption by **1**-EIR follows both the particle and pore diffusion models. Thus, the boundary layer mechanism could also be involved in the kinetics of Pd(II) sorption by **1**-EIR.

### Selective adsorption of Pd(II) from simulated mixed metal solution

Fig. S18 illustrates sorption behavior of **1**-EIR in the simulated solution containing 13 metals (Pd(II), Pt(IV), Rh(III), Al(III), Ba(II), Ce(III), Cu(II), Fe(III), La(III), Ni(II), Y(III), Zn(II), and Zr(IV)). **1**-EIR exhibited a high *S*% value for Pd(II) (97.5%), whereas those for the other metals were very low, especially that for Rh(III) (2.6%). This Pd(II) selectivity of **1**-EIR is attributed to the intriguing coordination environment of pincer-type extractant **1** and the relationship between the extractant and Pd(II) based on the hard–soft acid–base (HSAB) principle. Generally, Pd(II) prefers to adopt a square-planar geometry in HCl media, and the pincer-type extractant can provide a square-planar coordination environment for metal species. In addition, among the 13 metal species in solution, soft Pd(II) has a high affinity for the soft S donor based on the HSAB principle. Thus, the high Pd(II) selectivity of **1**-EIR is a results of the synergetic effect of these structural and chemical properties.

### Selective adsorption of Pd(II) from an automotive catalyst leachate

**Figure 7:**
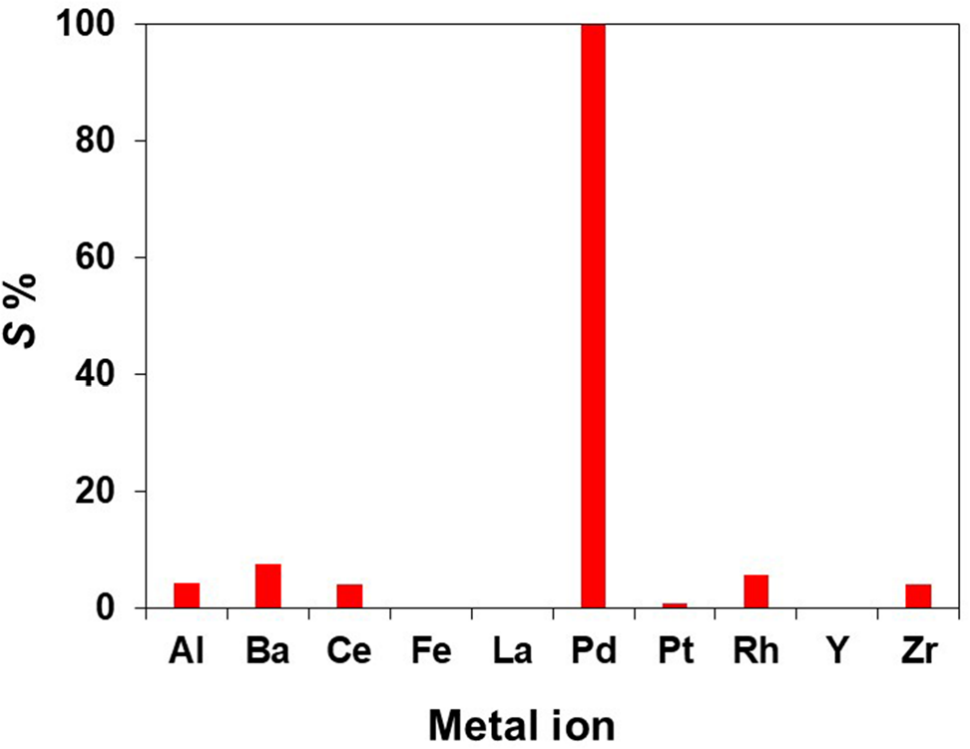
Sorption of Pd(II) from a spent automotive catalyst leachate containing 10 metals (Al, Ba, Ce, Fe, La, Pd, Pt, Rh, Y, and Zr) by **1**-EIR. Adsorption conditions: **1**-EIR = 0.1 g; pH = 0.5; shaking speed = 280 rpm; shaking time = 4 h.

Spent automotive catalysts, important secondary resources of PGMs, typically contain 1–2% PGMs and 90% supporting materials such as Al_2_O_3_, La_2_O_3_, CeO_2_, ZrO_2_, BaO, and other metal oxides. To demonstrate the applicability of the developed extractant system for the selective recovery of Pd(II) from secondary resources, **1**-EIR was applied to an automotive catalyst leachate, which contained 10 metals (Table S3). As shown in Fig. 7, **1**-EIR can adsorb Pd(II) selectively from the leachate. In particular, the *S*% value of Pd(II) was > 99.8%, whereas those of the other metals were less than 7.6%.

## Methods

### Materials

**Scheme 1:**
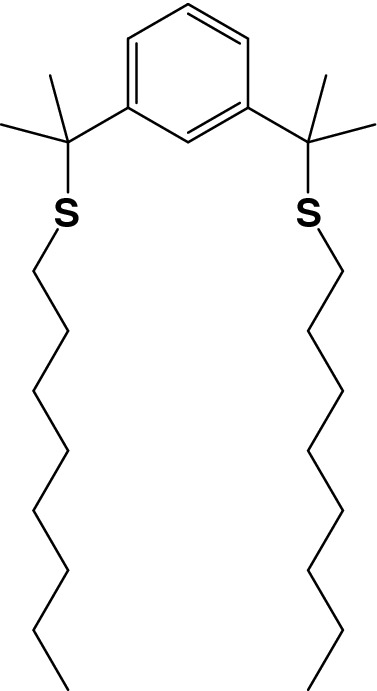
Structure of 1,3-Bis(2-(octylthio)propan-2-yl)benzene (**1**).

Pincer-type extractant **1** (Scheme 1) was synthesized according to a previous procedure^11^. Amberlite XAD-7 (Fig. S1 and Table S1), a synthetic resin, was obtained from Organo Corporation, Japan. Pd(II) stock solutions were prepared by dilution of a Pd standard solution (1000 ppm in 1 M HCl). A model solution included 13 metals (each at 100 mg L^−1^) was prepared by dissolving AlCl_3_, BaCl_2_, CeCl_3_∙7H_2_O, CuCl_2_, FeCl_3_, LaCl_3_∙7H_2_O, NiCl_2_, PdCl_2_, PtCl_4_, RhCl_3_, YCl_3_∙6H_2_O, ZnCl_2_, and ZrCl_4_ in 0.1 M HCl. Metal solutions at various concentrations were prepared by dissolving the required quantities of metal salts in doubly distilled water. All other chemicals were of reagent grade and used without further purification.

### Preparation of spent automotive catalyst leachate

The leachate was prepared according to a previous report^20^ and characterized by inductively coupled plasma atomic emission spectroscopy (ICP-AES).The leachate, which contained 10 metals (Al, Ba, Ce, Fe, La, Pd, Pt, Rh, Y, and Zr; Table S3), was used after 20-fold dilution with water (pH = 0.5).

### Preparation of 1-EIR

XAD-7 was washed with water, methanol, and acetone three times to remove salts and monomers. After filtration, the washed resin was dried under reduced pressure at 80 °C. Then, 0.5 g of XAD-7 was immersed in 25 mL of acetone containing 0.05–0.5 M of extractant **1** for 24 h at ambient temperature. Finally, the impregnated resin (**1**-EIR) was filtered and dried at 60 °C in vacuo for 3 h. The impregnated amount was calculated as follows:

9 $$ {\text{Impregnated}}\;{\text{amount}}\;({\text{mg}}\;{\text{g}}^{{ - {1}}} )\, = \,\left( {{\text{M}}_{i} - {\text{M}}_{e} } \right)/{\text{resin}}\;{\text{weight}} $$

where M_*i*_ and M_*e*_ are the amounts of **1** in acetone before and after impregnation.

### Pd(II) adsorption studies

For adsorption experiments, 0.1 g of **1**-EIR with 10 mL of stock Pd(II) solution (100 ppm) in 0.1–8.0 M HCl, simulated solution, or leachate in a 50 mL centrifuge tube was shaken at 280 rpm for 0–240 min. After filtration, the metal concentration in the aqueous phase was measured using ICP-AES (SPS-3000, Seiko Instruments Inc., Japan). The sorption capacity (*q* (mg g^−1^)) and sorption percentage (*S*%) were calculated as follows:

10 $$ q\, = \,\left[ {\left( {C_{0} \, - \,C_{e} } \right)/m} \right]\, \times \,V $$

11 $$ S\% \, = \,\left( {C_{0} \, - \,C_{e} /C_{0} } \right)\, \times \,{1}00 $$

where *C*_*0*_ and *C*_*e*_ are the metal concentrations in the aqueous phase before and after adsorption (mg L^−1^), *m* is resin weight (g), and *V* is the volume of metal solution (L).

### Desorption of Pd(II) from Pd(II)-loaded resin

For desorption, various desorption reagents (0.1 or 1.0 M thiourea (CH_4_N_2_S)/1.0 M HCl, 1.0 M CH_4_N_2_S/2.8 M NH_4_OH, 0.1 M NH_4_Cl/1.0 M HCl, 1.0 M Na_2_SO_3_/H_2_O, or 1.0 M Na_2_S_2_O_3_/H_2_O) were added to ~ 0.1 g of the Pd(II)-loaded **1**-EIR in a 50 mL centrifuge tube. After shaking at 280 rpm for 1 h and filtration, the metal concentration in the aqueous phase was measured using ICP-AES. The desorption percentage (*D*%) was calculated as follows:

12 $$ D\% \, = \,(C_{i} \, - \,C_{e} /C_{0} )\, \times \,{1}00 $$

where *C*_*i*_ is the initial amount of adsorbed Pd(II) (mg g^−1^) and *C*_*e*_ is the equilibrium amount of Pd(II) in the resin after recovery (mg g^−1^).

### Surface morphology analysis

The surface morphologies of the resins before and after treatment with Pd(II) were visualized by scanning electron microscopy (SEM) and energy-dispersive X-ray spectroscopy (EDX) using a Hitachi SU-70 Analytical UHR Schottky Emission Scanning Electron Microscope. The major and minor elements on the resin surface were determined qualitatively using X-ray photoelectron spectroscopy (XPS; Kratos Axis Ultra DLD) with a monochromatic Al Kα X-ray source (9.0 mA, 15 kV).

### ^1^H NMR analysis of adsorption mechanism

After Pd(II) adsorption by **1**-EIR, the Pd(II)–extractant **1** complex was flaked from the Pd(II)-loaded resin by extraction using chloroform. After solvent evaporation, the brown oil was redissolved in deuterated chloroform (CDCl_3_) and ^1^H NMR spectra were recorded (JEOL JNM-ECA 500). The chemical shifts (ppm) are relative to tetramethylsilane (TMS).

### Specific surface area and pore size distribution

Before vapor adsorption measurements, XAD-7 and **1**-EIR were pretreated at 100 °C at < 10^−3^ Torr for 1 day. The nitrogen adsorption isotherm of each resin (~ 100 g) was obtained at 77 K using a BELSORP-mini automated gas adsorption apparatus. The specific surface areas and pore size distributions of the resins were determined by the Brunauer–Emmett–Teller (BET) and Dollimore–Heal (DH) methods, respectively.

### Complete collection of Pd(II) via flaking

After the desorption process, 0.1 g of **1**-EIR with undesorbed Pd(II) was placed in an extraction thimble, inserted into a Soxhlet extractor, and refluxed with 150 mL of methanol for 12 h. After crushing the extracted resin, the presence of Pd(II) and extractant **1** in the resin was evaluated using XPS. The extracted Pd(II) solution from residual Pd(II) in **1**-EIR was measured by absorptiometric method using UV–Visible spectrophotometer (Shimazu UVmini-1240) at 311 nm.

## Conclusion

We demonstrated that **1**-EIR, composed of pincer-type extractant **1** impregnated in Amberlite XAD-7 resin, possesses a high affinity towards Pd(II) in solutions of only Pd(II), in a simulated mixed metal solution, and in a spent automotive catalyst leachate. Indeed, **1**-EIR efficiently adsorbed Pd(II), with a maximum sorption capacity of 49 mg g^−1^ achieved using a 700 ppm solution of Pd(II) in 0.1 M HCl after 24 h. Furthermore, a sorption percentage (*S*%) of > 99% was achieved for Pd(II) sorption by **1**-EIR in the spent automotive catalyst leachate containing 10 different metal ions, indicating the high Pd(II) selectivity of the developed EIR system. An examination of various sorption isotherm models suggested that Pd(II) sorption by **1**-EIR followed the Langmuir mechanism, which indicate that a single layer of Pd(II) was formed on **1**-EIR. The thermodynamic data showed that the Pd(II) sorption process was endothermic. The sorption dynamics based on reaction- and diffusion-based models suggested that Pd(II) adsorption by **1**-EIR followed the boundary layer mechanism, with adsorption occurring both on the surface and in the pores of **1**-EIR. This mechanism was also supported by the ^1^H NMR analysis of the complex flaked from Pd(II)-loaded **1**-EIR. Furthermore, Pd(II) desorption from Pd(II)-loaded **1**-EIR was achieved using a CH_4_N_2_S/HCl as the desorption reagent, and the resin could be reused effectively over 20 cycles. The complete stripping of any Pd(II) remaining in Pd(II)-loaded **1**-EIR after the desorption process was possible via Soxhlet extraction of the Pd(II)–extractant complex. These findings will significantly contribute to the further development of environmentally friendly adsorption techniques that avoid the use of harmful organic diluents to separate Pd from the leachates of secondary resources or industrial effluents.

## Supplementary Information

Supplementary Information
