# Supplementary material for: Environmentally friendly Pd(II) recovery from spent automotive catalysts using resins impregnated with a pincer-type extractant
Source: Sci Rep. 2021 Jan 11;11:365. doi: 10.1038/s41598-020-79614-2 (PMC7801578; doi:10.1038/s41598-020-79614-2)
Supplement: Supplementary file 1 — Supplementary Information [file 41598_2020_79614_MOESM1_ESM.docx]

**Supplementary Information**

**Environmentally Friendly Pd(II) Recovery from Spent Automotive Catalysts Using Resins Impregnated with a Pincer-Type Extractant**

Manabu Yamada^1^, Shun Kimura^2^, Muniyappan Rajiv Gandhi^3^ & Atsushi Shibayama^4^

^1^ Research Center of Advanced Materials for Breakthrough Technology, Graduate School of Engineering Science, Akita University, 1-1 Tegatagakuen-machi, Akita 010-8502, Japan; ^2^Applied Chemistry Course, Department of Materials Science, Graduate School of Engineering Science, Akita University, 1-1 Tegatagakuen-machi, Akita 010-8502, Japan; ^3^ Quality Control Department, Panipat Refinery & Petrochemical Complex, Indian Oil Corporation Limited, Haryana, 132140, India; ^4^ Graduate School of International Resources Science, Akita University, 1-1 Tegatagakuen-machi, Akita 010-8502, Japan

*Corresponding author. Tel +81 18 889 3068; fax: +81 18 889 3068.

E-mail address: myamada@gipc.akita-u.ac.jp

# Table of content

**Table S1.** Physical and chemical properties of XAD resin.

**Figure S1.** (a) The chemical structure of XAD-7 and (b) cluster model of XAD-7 surface.

**Figure S2.** The relationship between impregnated amounts of **1** into XAD-7 and Pd(II) quantity adsorbed by **1**-EIR. Sorption condition: [HCl] = 0.1~8.0 M, [Pd(II)] = 100 ppm (= mg L^−1^), **1**-EIR = 0.1 g, shaking speed = 280 rpm, shaking time = 24 h.

**Figure S3.** Effect of shaking time for Pd(II) adsorption capability of **1**-EIR. Sorption condition: [HCl] = 0.1 M, [Pd(II)] = 100 ppm (= mg L^−1^), **1**-EIR = 0.1 g, shaking speed = 280 rpm, shaking time = 0 ~ 240 min.

**Figure S4.** Photographs a) before and b) after Pd(II) adsorption by **1**-EIR.

**Figure S5.** Nitrogen isotherms of XAD-7 (red circle) and **1**-EIR (blue circle).

**Figure S6.** Brunauer-Emmett-Teller (BET) plots and the sorption parameters of a) XAD-7 and b) **1**-EIR.

**Figure S7.** Pore size distribution of XAD-7 (red circle) and **1**-EIR (blue circle) by Dollimore-Heal (DH) method.

**Figure S8.** The sizes of a PdCl_4_^2-^ species and a CH_4_N_2_S molecule.

**Figure S9.** Photographs a) before and b) after Soxhlet extraction of residual Pd(II) in **1**-EIR.

**Figure S10.** Full scan X-ray photoelectron spectroscopy (XPS) after Soxhlet extraction from the undesorbed Pd(II) in **1**-EIR.

**Figure S11.** ^1^H NMR spectra of the Pd(II)–extractant **1** complex flaked from Pd(II)-loaded **1**-EIR.

**Figure S12.** a) Freundlich and b) Langmuir isotherms for Pd(II) sorption by **1**-EIR. Sorption conditions: **1**-EIR = 0.1 g, [HCl] = 0.1 M; [Pd(II)] = 500–700 ppm (= mg L^−1^); temperature = 293–313 K; shaking time = 24 h; shaking speed = 80 rpm.

**Figure S13.** Van’t Hoff plot relating ln *K_0_* against 1/T for Pd(II) sorption by **1**-EIR. Sorption conditions: **1**-EIR = 0.1 g, [HCl] = 0.1 M; [Pd(II)] = 500–700 ppm (= mg L^−1^); temperature = 293–313 K; shaking time = 24 h; shaking speed = 80 rpm.

**Figure S14.** Pseudo-first-order for Pd(II) sorption by **1**-EIR. Sorption condition: [HCl] = 0.1 M, [Pd(II)] = 600 ppm (= mg L^−1^), **1**-EIR = 0.1 g, shaking speed = 280 rpm, shaking time = 0.5~12 h.

**Figure S15.** Pseudo-second-order for Pd(II) sorption by **1**-EIR. Sorption condition: [HCl] = 0.1 M, [Pd(II)] = 600 ppm (= mg L^−1^), **1**-EIR = 0.1 g, shaking speed = 280 rpm, shaking time = 0.5~12 h.

**Figure S16.** Particle diffusion for Pd(II) sorption by **1**-EIR. Sorption condition: [HCl] = 0.1 M, [Pd(II)] = 600 ppm (= mg L^−1^), **1**-EIR = 0.1 g, shaking speed = 280 rpm, shaking time = 0.5~12 h.

**Figure S17.** Intraparticle diffusion for Pd(II) sorption by **1**-EIR. Sorption condition: [HCl] = 0.1 M, [Pd(II)] = 600 ppm (= mg L^−1^), **1**-EIR = 0.1 g, shaking speed = 280 rpm, shaking time = 0.5~12 h..

**Table S2.** Application of kinetic models to the sorption of Pd(II) ions onto **1**-EIR at r.t.

**Figure S18.** Selective Pd(II) sorption of **1**-EIR from a simulated 13 metals containing HCl solution. Sorption condition: [HCl] = 0.1 M, [Metals] = each 100 ppm (= mg L^−1^), **1**-EIR = 0.1 g, shaking speed = 280 rpm, shaking time = 3 h.

**Table S3.** Metal species and metal concentration in the leachate of automotive catalyst.

**Table S1.** Physical and chemical properties of XAD resin.

| **Constituents** | |
| --- | --- |
| Matrix structure | Acrylic |
| Classification | Nonionic Spherical |
| Specific surface area (m²/g) | 500 |
| Pore ​​volume (cm³/g) | 0.6 |
| Pore diameter (Å) | 100 |
| Apparent density (g/L) | ~655 |
| Moisture holding capacity (%) | 61-69 |
| Harmonic mean diameter (mm) | 0.43 - 0.69 |
| Uniformity coefficient | ≦ 2.0 |
| Effective pH range | 0-14 |
| Polarity | Intermediate polarity |

**Figure S1.** (a) The chemical structure of XAD and (b) cluster model of XAD surface.

**Figure S2.** The relationship between impregnated amounts of **1** into XAD-7 and Pd(II) quantity adsorbed by **1**-EIR. Adsorption condition: [HCl] = 0.1~8.0 M, [Pd(II)] = 100 ppm (= mg L^−1^), **1**-EIR = 0.1 g, shaking speed = 280 rpm, shaking time = 24 h.

**Figure S3.** Effect of shaking time for Pd(II) adsorption capability of **1**-EIR. Sorption condition: [HCl] = 0.1 M, [Pd(II)] = 100 ppm (= mg L^−1^) , **1**-EIR = 0.1 g, shaking speed = 280 rpm, shaking time = 0 ~ 240 min.


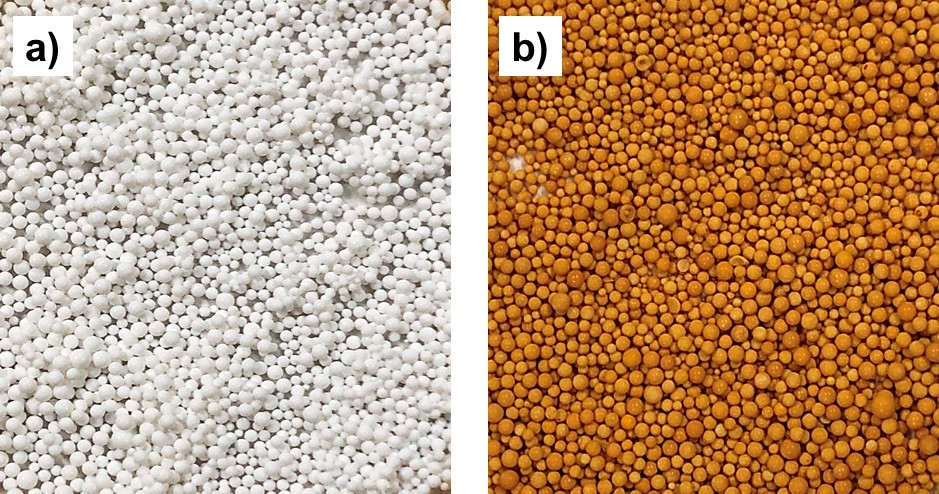


**Figure S4.** Photographs a) before and b) after Pd(II) adsorption by **1**-EIR.

**Figure S5.** Nitrogen isotherms of XAD-7 (red circle) and **1**-EIR (blue circle).

**
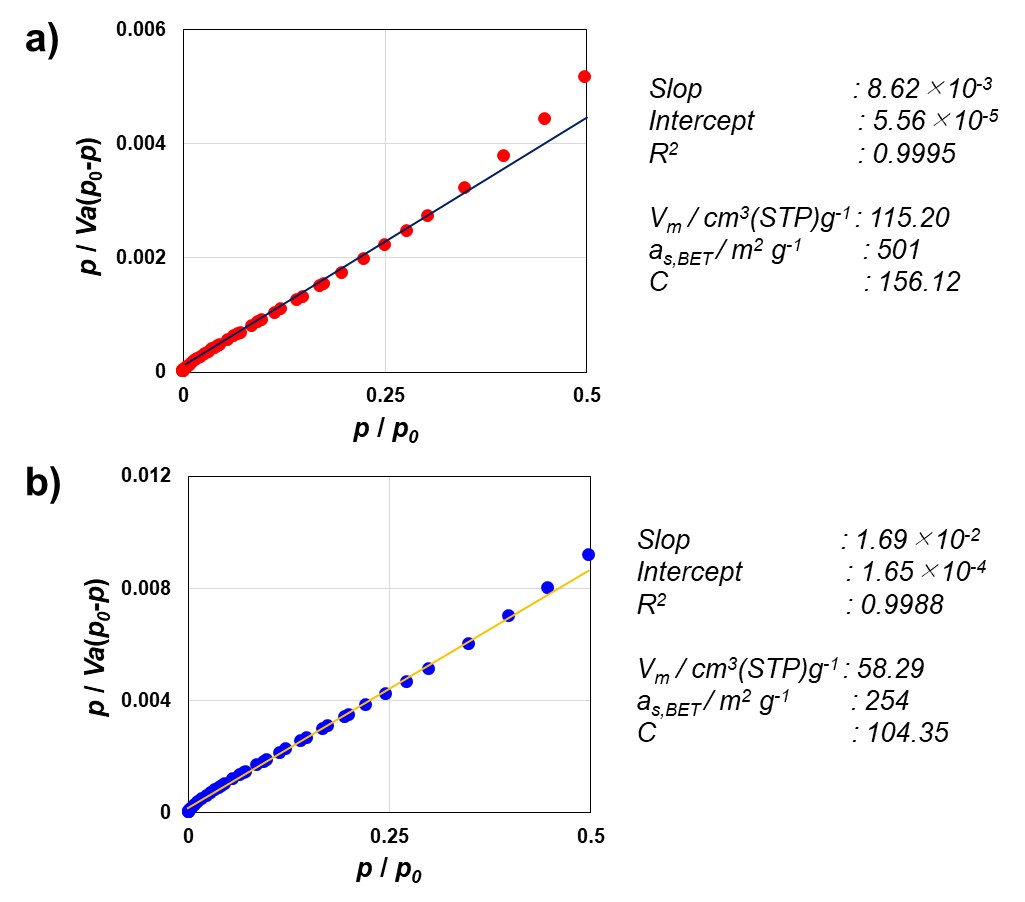
**

**Figure S6.** Brunauer-Emmett-Teller **(**BET) plots and the sorption parameters of a) XAD-7 and b) **1**-EIR.

**Figure S7.**  Pore size distribution of XAD-7 (red circle) and **1**-EIR (blue circle) by Dollimore-Heal (DH) method.

**Figure S8.** The sizes of a PdCl_4_^2-^ species and a CH_4_N_2_S molecule.


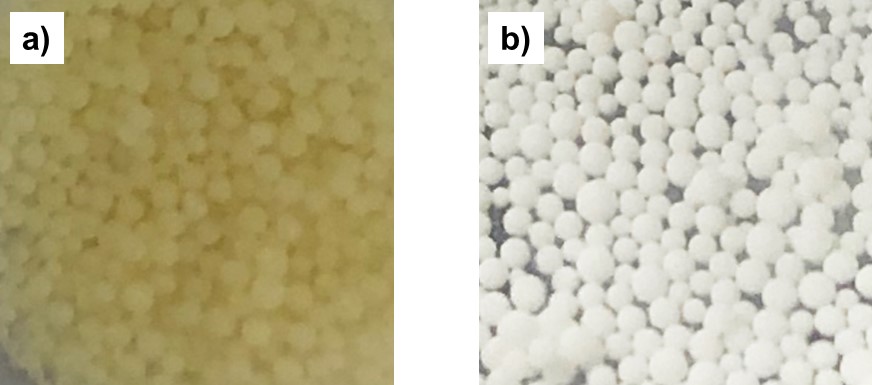


**Figure S9.** Photographs a) before and b) after Soxhlet extraction of residual Pd(II) in **1**-EIR.


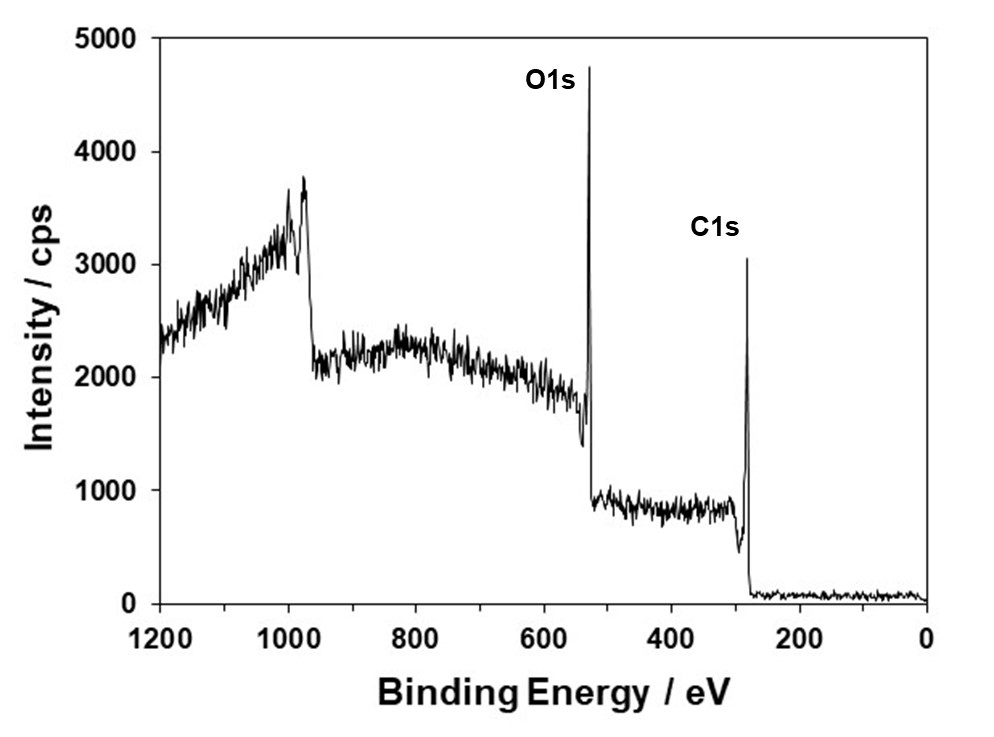


**Figure S10.** Full scan X-ray photoelectron spectroscopy (XPS) after Soxhlet extraction from the undesorbed Pd(II) in **1**-EIR.


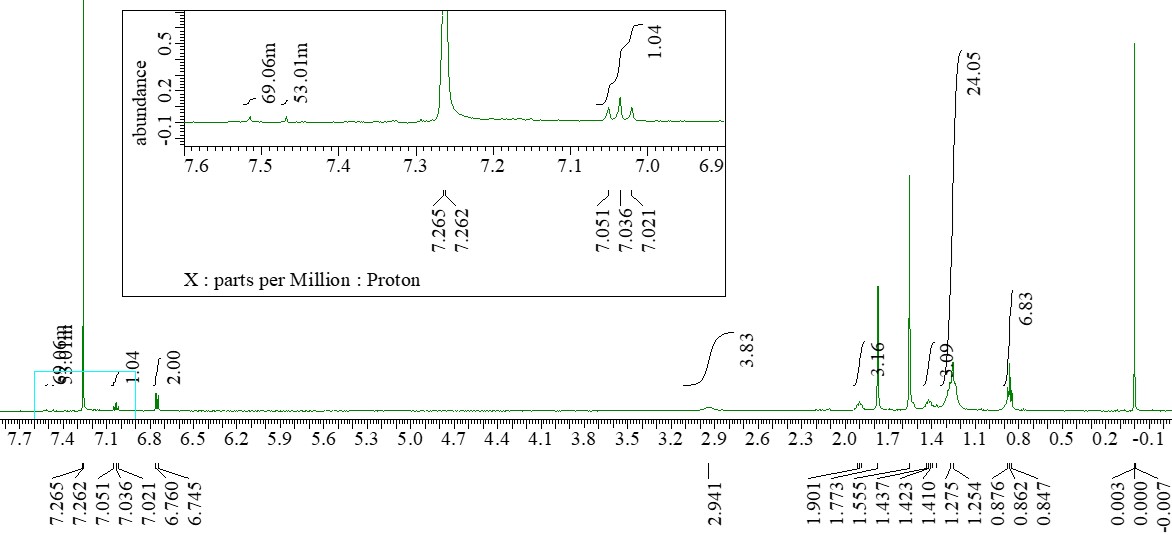


**Figure S11.** ^1^H NMR spectra of the Pd(II)–extractant **1** complex flaked from Pd(II)-loaded **1**-EIR.

**Figure S12.** a) Freundlich and b) Langmuir isotherms for Pd(II) sorption by **1**-EIR. Sorption conditions: **1**-EIR = 0.1 g, [HCl] = 0.1 M; [Pd(II)] = 500–700 ppm (= mg L^−1^); temperature = 293–313 K; shaking time = 24 h; shaking speed = 80 rpm.

**Figure S13.** Van’t Hoff plot relating ln *K_0_* against 1/T for Pd(II) sorption by **1**-EIR. Sorption conditions: **1**-EIR = 0.1 g, [HCl] = 0.1 M; [Pd(II)] = 500–700 ppm (= mg L^−1^); temperature = 293–313 K; shaking time = 24 h; shaking speed = 80 rpm.

**Figure S14.** Pseudo-first-order for Pd(II) sorption by **1**-EIR. Sorption condition: [HCl] = 0.1 M, [Pd(II)] = 600 ppm (= mg L^−1^), **1**-EIR = 0.1 g, shaking speed = 280 rpm, shaking time = 0.5~12 h.

**Figure S15.** Pseudo-second-order for Pd(II) sorption by **1**-EIR. Sorption condition: [HCl] = 0.1 M, [Pd(II)] = 600 ppm (= mg L^−1^), **1**-EIR = 0.1 g, shaking speed = 280 rpm, shaking time = 0.5~12 h.

**Figure S16.** Particle diffusion for Pd(II) sorption by **1**-EIR. Sorption condition: [HCl] = 0.1 M, [Pd(II)] = 600 ppm (= mg L^−1^), **1**-EIR = 0.1 g, shaking speed = 280 rpm, shaking time = 0.5~12 h.

.

**Figure S17.** Intraparticle diffusion for Pd(II) sorption by **1**-EIR. Sorption condition: [HCl] = 0.1 M, [Pd(II)] = 600 ppm (= mg L^−1^), **1**-EIR = 0.1 g, shaking speed = 280 rpm, shaking time = 0.5~12 h.

**Table S2.** Application of kinetic models to the sorption of Pd(II) ions onto **1**-EIR at r.t.

| Kinetic models | Parameters | Pd(II) = 600 ppm  **1**-EIR |
| --- | --- | --- |
| Pseudo-first-order | *k_ad_* (min^-1^) | 5.0E -4 |
|  | *r* | 0.8650 |
| Pseudo-second-order | *q_e_* (mg/g) | 25.00 |
|  | *k* (g/mg min) | 3.163E -4 |
|  | *h* (mg/g min) | 0.2164 |
|  | *r* | 0.9985 |
| Particle diffusion | *k_p_* (mg/g min) | 5.9E -3 |
|  | *r* | 0.9999 |
| Intraparticle diffusion | *k_i_* (mg/g min^0.5^) | 0.8773 |
|  | *r* | 0.9938 |

**Figure S18.** Selective Pd(II) sorption of **1**-EIR from a simulated 13 metals containing HCl solution. Sorption condition: [HCl] = 0.1 M, [Metals] = each 100 ppm (= mg L^−1^), **1**-EIR = 0.1 g, shaking speed = 280 rpm, shaking time = 3 h.

**Table S3.** Metal species and metal concentration in the leachate of automotive catalyst

| Metal ions | [M]_aq,init_ (mg/L) |
| --- | --- |
| Pd(II)  Pt(IV)  Rh(III)  La(III)  Ce(III)  Y(III)  Zr(IV)  Ba(II)  Al(III)  Fe(III) | 111.4  53.6  41.4  84.4  569.1  4.0  19.8  307.7  291.5  7.7 |
